# Identification of Adverse Drug Event–Related Japanese Articles: Natural Language Processing Analysis

**DOI:** 10.2196/22661

**Published:** 2020-11-27

**Authors:** Shogo Ujiie, Shuntaro Yada, Shoko Wakamiya, Eiji Aramaki

**Affiliations:** 1 Nara Institute of Science and Technology Nara Japan

**Keywords:** adverse drug events, medical informatics, natural language processing, pharmacovigilance

## Abstract

**Background:**

Medical articles covering adverse drug events (ADEs) are systematically reported by pharmaceutical companies for drug safety information purposes. Although policies governing reporting to regulatory bodies vary among countries and regions, all medical article reporting may be categorized as precision or recall based. Recall-based reporting, which is implemented in Japan, requires the reporting of any possible ADE. Therefore, recall-based reporting can introduce numerous false negatives or substantial amounts of noise, a problem that is difficult to address using limited manual labor.

**Objective:**

Our aim was to develop an automated system that could identify ADE-related medical articles, support recall-based reporting, and alleviate manual labor in Japanese pharmaceutical companies.

**Methods:**

Using medical articles as input, our system based on natural language processing applies document-level classification to extract articles containing ADEs (replacing manual labor in the first screening) and sentence-level classification to extract sentences within those articles that imply ADEs (thus supporting experts in the second screening). We used 509 Japanese medical articles annotated by a medical engineer to evaluate the performance of the proposed system.

**Results:**

Document-level classification yielded an F1 of 0.903. Sentence-level classification yielded an F1 of 0.413. These were averages of fivefold cross-validations.

**Conclusions:**

A simple automated system may alleviate the manual labor involved in screening drug safety–related medical articles in pharmaceutical companies. After improving the accuracy of the sentence-level classification by considering a wider context, we intend to apply this system toward real-world postmarketing surveillance.

## Introduction

### Background

According to the World Health Organization, an adverse drug event (ADE) is any untoward occurrence that may present during treatment with a pharmaceutical product but is not necessarily causally related to the treatment [1]. According to a survey conducted by Howard et al [2], ADEs are responsible for approximately 3.7% of all hospital admissions worldwide. This issue has been addressed by institutional premarketing and postmarketing drug safety surveillance. However, postmarketing measures play a more important role than premarketing clinical trials, as postmarketing measures can also detect infrequent reactions, long-term effects, and drug–drug/drug–food interactions [3]. A major source of postmarketing surveillance is spontaneous or voluntary reporting of suspected adverse reactions by clinicians, pharmacists, and the pharmaceutical industry. However, owing to the high volume of incoming “signals,” the identification of even a few credible reports is labor-intensive [4]. Hence, the development of an automated system that determines and classifies the relative importance of clinical ADE-related reports would be considerably beneficial.

Existing automation research targets different source materials, reflecting the wide range of signals processed by real-world postmarketing surveillance. These inputs include electronic health records [5,6], patient reports [7], medical articles [8,9], and social media posts [10,11]. This study focuses on medical articles as they comprise a substantial portion of postmarketing surveillance in many countries. Pharmaceutical companies voluntarily send reports based on medical articles to regulatory bodies [4].

Policies governing the reporting of ADEs to regulatory bodies vary among countries and regions [12]. In general, reporting may be precision or recall based. In the former approach, implemented in the United States [13] and the European Union [14], suspected serious adverse drug reactions (ADRs) are rapidly reported. Serious ADRs correspond to certain ADEs for which reasonable causal relationships between events and drugs are suspected or confirmed. In recall-based reporting, any possible ADE must be reported immediately. ADEs include cases where a causal relationship between drugs and harmful events cannot be ruled out [15]. Nevertheless, this strategy can introduce numerous false negatives or substantial amounts of noise. Processing a large volume of reports on all possible ADEs greatly increases the manual classification burden. Overall, recall-based reporting is very difficult to accomplish using limited human labor.

Japan has adopted and implemented recall-based pharmacovigilance [15]. Its main information source is spontaneous reporting from pharmaceutical companies, and the basis of these reports is medical articles. This process usually consists of a first (initial) screening followed by a second screening. In the first screening, medical articles are manually classified and prioritized. For example, if an article mentions fatal or lethal ADEs, it receives top priority. The second in-depth screening is performed by medical experts and assesses the merit of the reported ADEs. In the Japanese pharmaceutical company involved in this survey, thousands of articles must be monitored annually, and each report requires at least 10 minutes to evaluate. This process incurs a significant labor cost. Moreover, the criteria used in the first screening may be subjective (and thus vary considerably according to the person conducting it). Consequently, the surveillance process may be unnecessarily delayed.

### Objectives

To address Japanese pharmacovigilance, we have developed an automated system that replaced the first screening by extracting ADE-containing articles. For the second screening, we also enlisted the services of medical experts to identify ADE-suggesting sentences in the articles. Our system combines both document- and sentence-level classification models. It classifies Japanese medical articles to extract those that contain references to ADEs and then uses them as candidates for the second screening (*ADE-containing article extraction*). It also classifies the ADE-suggesting sentences that must be scrutinized by medical experts (*ADE-suggesting sentence extraction*).

To this end, we implemented natural language processing (NLP) techniques. Our system consists of simple machine learning methods that are easily applied and managed in-house by pharmaceutical companies. Targeting Japanese medical articles also offers insights into an effective management approach for papers written in non-English languages with few linguistic resources within the medical domain.

- To support postmarketing surveillance in Japan where recall-based reporting is adopted for drug safety, we built an automated system identifying ADE-containing medical articles and the ADE-suggesting sentences therein to improve interpretability.
- Our proposed models classified the ADE-containing articles at a 0.903 F1 score and ADE-suggesting sentences at a 0.413 F1 score based on a manually annotated test set of Japanese medical articles.
- We developed an effective automated system based on relatively simple models. It can be easily implemented and managed in-house by pharmaceutical companies. In addition, our system may be readily expanded to classify papers written in non-English languages.

## Methods

### Materials

Japanese medical articles used for postmarketing surveillance duty in a Japanese pharmaceutical company were provided for subsequent analysis. The majority of the articles were related to a select range of drugs surveyed by the company, but were not limited to specific clinical areas or diseases. The frequency with which each drug appears in the data is reported in Multimedia Appendix 1. The articles were randomly sampled, and text data were extracted from PDF documents by optical character recognition (OCR) using WinReader PRO version 15.0 (NTTDATA NJK Corporation) [16]. Articles written in 2 or more columns were excluded as the OCR software had difficulty recognizing text in this format. Subsequent filtering generated 509 medical articles. Certain symbols such as “$”and “^” that do not normally appear in Japanese medical articles were removed.

After preprocessing, all sentences were filtered on the basis of the appearance of ADEs. These were judged according to the following criteria:

1. An adverse event was mentioned after a drug prescription; or
2. The author explicitly mentioned the occurrence of a suspicious ADE.

Matched sentences were labeled as *ADE suggesting*. This determination was made by considering multiple sentences. Hence, the ADE-suggesting designation may have spanned several sentences.

Medical articles containing any ADE-suggesting sentences were designated *ADE containing*. Here, 300/509 articles (58.9%) were labeled ADE containing. Table 1 shows the average number of sentences and characters per medical article in the respective ADE-containing articles.

**Table 1:** Average number of sentences and characters per medical article.

Label		Number of sentences	Number of characters
**ADE^a^ suggesting, mean (SD)**			
	All	3.9 (2.7)	321.7 (456.1)
	Criterion A	3.5 (2.6)	399.3 (283.9)
	Criterion B	0.4 (0.7)	56.8 (112.3)
Non-ADE suggesting, mean (SD)		48.2 (72.1)	2897.0 (4104.0)

^a^ADE: adverse drug event.

The corpus was annotated by a medical engineer. To evaluate annotation quality, Cohen κ [17] was calculated using parallel annotation data generated by the medical engineer and an author with no prior medical experience. The latter person separately annotated 51/509 (10.0%) of the data set. The Cohen κ values were 0.638 for sentence-level annotation and 0.841 for document-level annotation in the 51/509 (10.0%) sample. Both of these satisfied the standard quality criterion for computational linguistic corpora. Thus, the entire annotation prepared by the medical engineer was adopted for this study.

### System Architecture

**Figure 1:**
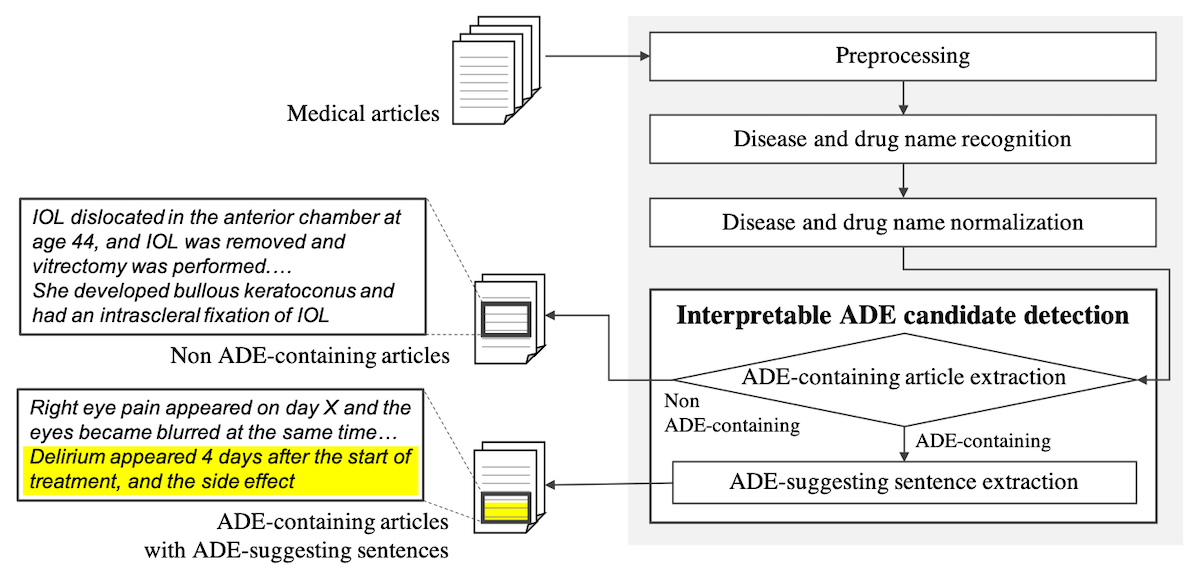
Architecture of the proposed system. ADE: adverse drug event; IOL: intraocular lens.

Figure 1 presents an overview of the architecture of the proposed system. The system comprised preprocessing, disease and drug name recognition, disease and drug name normalization, and interpretable ADE candidate detection submodules.

1. Preprocessing: Sentences were automatically separated by Japanese full stops and periods only after Japanese letters (including hiragana, katakana, and Chinese characters).
2. Disease and drug name recognition: Disease and drug names were extracted from preprocessed articles. Disease names were extracted with MedEX/J , a disease name extractor provided by Ito et al.[18] which uses conditional random fields (CRFs). This technique is commonly used for named entity recognition and is trained on Japanese case reports. It should be noted that MedEX/J can also extract any English disease names occurring in Japanese medical articles. Drug names were extracted with CRF trained in the same way as MedEX/J. Articles were fed into the models by converting them into the character-based IOB2 format widely used for named entity recognition.
3. Disease and drug name normalization: As there are many variations on the same disease and drug names, they were normalized with the MANBYO [19] and HYAKUYAKU [20] dictionaries. The MANBYO dictionary, the largest Japanese disease name dictionary, can link more than 300,000 disease names (as of September 2020) extracted from Japanese medical documents to various standard forms, such as MedDRA, ICD-10, ICD-11. The HYAKUYAKU dictionary holds more than 40,000 drug names (as of September 2020) extracted from Japanese medical documents and questionnaires to patients and is linked to generic names. These dictionaries also contain English disease/drug names appearing in Japanese medical documents, which can also be normalized.
  Distance-based similarity was edited to normalize disease and drug names [21] between extracted and dictionary words. The extracted word was then replaced in the standard expression linked to the dictionary word with the highest similarity. Extracted words were not replaced when they had no dictionary words with similarity exceeding the 0.3 threshold set in this system on the basis of a preliminary experiment.
4. Interpretable ADE candidate detection: This was performed using normalized disease and drug names as features and extracting candidate articles related to ADEs with ADE-suggesting sentences for the second screening.

### Interpretable ADE Candidate Detection

#### Overview

Interpretable ADE candidate detection was conducted to extract useful information for the second screening. ADE-containing article extractions and ADE-suggesting sentence extractions were both performed. Both extractions use binary-classification models. In ADE-containing article extraction, the articles were classified as “ADE containing” or “non-ADE containing.” The sentences in “ADE-containing” articles were then classified as “ADE suggesting” or “non-ADE suggesting” in ADE-suggesting sentence extraction. Several design aspects of the system, including the classification algorithm and the feature design used in each model, are described below.

#### Classification Algorithm

Logistic regression was used to classify the articles and sentences. This method has been widely implemented for text classification. Neural network (NN) models usually outperform other machine learning–based models such as logistic regression in many NLP tasks. However, NN models require much larger corpora and their output is harder to interpret [22]. By contrast, logistic regression can be trained using comparatively less annotated data and the contribution of each feature is easy to determine. Therefore, logistic regression rather NN models was adopted here. The LogisticRegression class of scikit-learn [23] was applied with its default parameters.

#### Feature Design

An earlier study [24] used the assumption that each sentence refers to at least one disease and drug, and subsequently used identifying features in the words surrounding these key words. Here, however, it was assumed that each sentence does not necessarily refer to any disease or drug. Thus, certain statistical features were created for the setting used here.

For ADE-containing article extraction, expressions alluding to an ADE such as “We stopped the drug” were regarded as important clues for detecting ADEs. The starting point was text in articles as features in orthodox Bag-of-Words representations. MeCab was used to create this Bag-of-Words feature [25]. MeCab is a Japanese morphological analyzer used to separate sentences into words. Those words that appeared only once were removed.

Features concerning diseases and drugs were considered useful for ADE detection as they played key roles in manual ADE detection. Therefore, standard expressions and the sum of their frequency were used as features to account for individual disease and drug characteristics.

To extract ADE-suggesting sentences, the context needs to be considered (as “ADE suggesting” may span multiple sentences). Thus, the features of previous and post sentences in ADE-suggesting sentence extraction, and the same features as ADE-containing article extraction were used. The feature set of interpretable ADE candidate detection is listed below.

- Word tokens: Bag of words appearing in text;
- Standard disease/drug name: Bag of standard disease and drug names;
- Sum of disease/drug name: Sum of occurrence of disease names and sum of occurrence of drug names;
- Context word tokens: Bag of words in previous and post sentences;
- Context standard disease/drug name: Bag of standard disease and drug names in previous and post sentences;
- Context sum of disease/drug name: Sum of occurrence of disease names and sum of occurrence of drug names in previous and post sentences.

The features of each model are shown in Table 2.

**Table 2:** Feature set used in ADE-containing article extractions and ADE-suggesting sentence extractions.^a^

Feature	ADE^b^-containing article extraction	ADE-suggesting sentence extraction
Word tokens	✓ (7188)	✓ (6597)
Standard disease/drug name	✓ (1043)	✓ (1083)
Sum of disease/drug name	✓ (2)	✓ (2)
Context word tokens	X	✓ (13,194)
Context standard disease/drug name	X	✓ (2166)
Context sum of disease/drug name	X	✓ (4)

^a^The figures in parentheses indicate the average number of variables.

^b^ADE: adverse drug event.

### Experiments

#### Setting

Experiments were conducted to evaluate ADE-containing article extractions and ADE-suggesting sentence extractions.

For ADE-containing article extraction, the classifier trained and predicted the articles by fivefold cross-validation using the features listed in Table 3. All 5 splits of the articles were randomly sampled with the label proportion kept.

**Table 3:** Effects of each feature in adverse drug event–containing article extraction.

Feature	ΔF1 score
Without word tokens	–0.0456
Without standard disease/drug name	–0.0001
Without sum of disease/drug name	–0.0155

For ADE-suggesting sentence extraction, fivefold cross-validation was applied at the document level. Articles labeled “non-ADE containing” lack sentences labeled “ADE suggesting.” Hence, the label proportion was unbalanced when all of the articles in the training set were used for training. To avoid this disequilibrium, only sentences in the “ADE-containing” articles were used for training and all sentences in the test set were used for evaluation.

#### Evaluation Metrics

Based on the experimental results, F1 scores were calculated to evaluate the performance of our models. To analyze the performance more precisely, we also made precision–recall curves. A precision–recall curve plots recall and precision at each threshold and evaluates tasks with significant trade-offs between measures. High recall or sensitivity means that a model misses no ADEs. This feature is critical for ADE detection. High precision means that the model prediction is reliable. Thus, we must detect ADEs with both reasonable precision and high recall.

## Results

### Performance

The average cross-validation for ADE-containing article extraction yielded F1 = 0.903 (SD 0.0165). For ADE-suggesting sentence extraction, F1 was substantially lower; F1 = 0.413 (SD 0.0247).

**Figure 2:**
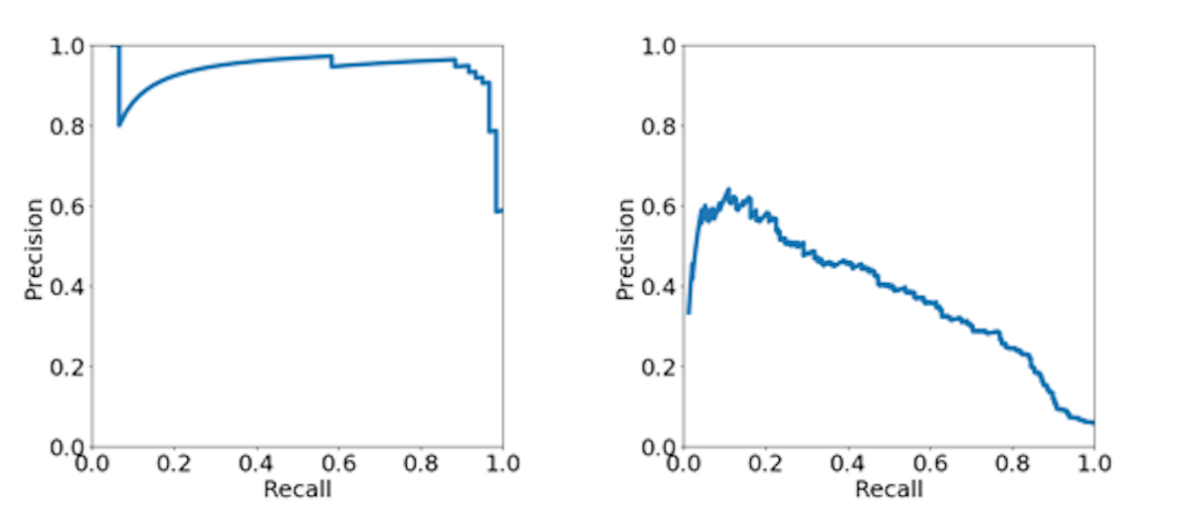
Precision-recall curves for (a) ADE-containing article extraction and (b) ADE-suggesting sentence extraction. ADE: adverse drug event.

The precision–recall curves for the validation set with the highest F1 are shown in Figure 2. For ADE-containing article extraction, we achieved high precision and recall. By contrast, both precision and recall were relatively low for ADE-suggesting sentence extraction.

### Feature Analysis

We conducted an ablation study, which is an evaluation method that removes each feature to quantify the effects of that feature. We removed each feature group and obtained an average ߡF1 score.

Tables 3 and 4 show the results of the ablation studies performed on ADE-containing article extractions and ADE-suggesting sentence extractions in each model. For both models, the Bag-of-Words feature in the corresponding sentence (word tokens) contributed the most. The standard disease and drug names had no influence on classification performance (Table 4). Likewise, the contextual features of standard disease and drug names had no influence on classification performance (Table 4).

**Table 4:** Effects of each feature in adverse drug event–suggesting sentence extraction.

Feature	ΔF1 score
Without word tokens	–0.0644
Without context word tokens	0.0070
Without standard disease/drug name	0.0
Without context standard disease/drug name	0.0
Without sum of disease/drug name	–0.0204
Without context sum of disease/drug name	–0.0012

## Discussion

### Feasibility of the Proposed Approach

#### Performance

The objective of this study was to build a system that supports Japanese drug postmarketing surveillance by automating the first screening and supporting the second screening with medical expertise. Our system effectively addressed this by dividing the task into a relatively easy task, namely, detecting ADEs at the document level, and a comparatively difficult one, that is, detecting ADEs at the sentence level.

Our system classified medical articles related to ADEs with high precision and recall. This result suggests that complex models such as relation classification are unnecessary for this application. Rather, simple document classification suffices to replace manual work in the first screening and thus reduce annotation costs.

The performance of our classification system for ADE-suggesting sentence extraction was relatively poor. However, from the viewpoint of our original goal (supporting experts in drug safety monitoring), performance at this level would still save a large amount of time and cost. Thus, in cases where the model classifies sentences with high recall, there is a high chance of an expert finding the ADE-suggesting sentence after a comparatively short search. In addition, our system was competitive with respect to other relation classification models that extract and classify diseases and drugs according to the relationships among them. The overall performance of ADE-drug relation-based classification is approximately 40%-60% [6].

#### Feature Contribution

In terms of the feature contribution, word tokens are the features that contributed the most to classify ADE-containing articles and ADE-suggesting sentences. By contrast, the standard disease and drug names and contextual features had less influence on classification performance compared to word tokens. This indicates that the extraction of disease and drug names, which requires relatively large training data to build models, is not necessarily needed to maintain accuracy. All features with their coefficients for ADE-containing article extraction and ADE-suggesting sentence extraction are listed in Multimedia Appendices 2 and 3.

We assumed that language-dependent features such as word embeddings, which are vector representations commonly used to consider the semantics of words, would potentially improve performance. However, obtaining high-quality word embeddings require numerous raw texts, and would be hard to prepare in languages other than English (especially in the medical domain). Thus, we focused more on using language-independent features. The features of each model do not depend on the characteristics of the Japanese language. Therefore, our system is readily applicable to papers written in non-English languages that have relatively small annotated corpora.

### Error Analysis

We achieved relatively poor performance for ADE-suggesting sentence extraction. Therefore, we investigated the classification errors of the ADE-suggesting sentence extraction model and used all features for qualitative system output analysis.

Table 5 shows examples of the system classification. For each example, the first sentence is the previous one and the remainder is the corresponding sentence. Note that the terms “gold standard” and “system prediction” represent the corresponding sentence labels. In this section, we analyze cases (c)–(f) that were misclassified by the system.

**Table 5:** Examples of classification results.

Case	True label	Prediction	Sentences
(a)	ADE^a^	ADE	MTX^b^ + adalimumab administration started. Changed to certolizumab pegol because of MTX’s side effects.
(b)	ADE	ADE	Case: Male, 74 years old. [Previous history] Rash due to ABPC/SBT^c^. Anaphylactic shock at CEZ^d^.
(c)	ADE	Non-ADE	MTX was administered to a 59-year-old patient with RA^e^. In March 201X, she had difficulty breathing and was consulted.
(d)	ADE	Non-ADE	Case: Female, age 79 years. [Chief complaint] [Current medical history] Patient was taking prednisolone 40 mg/d and methotrexate 8 mg/wk. The patient presented with giant cell arteritis and was using insulin for diabetes. Two weeks later, she was hospitalized for malaise and poor appetite.
(e)	Non-ADE^f^	ADE	Stevens–Johnson syndrome (SJS) is characterized by fever and severe mucosal eruptions of the skin, mucosal transitions involving the eyes, lips, and vulva, and blister and erosion due to erythema and necrotic injury to the epidermis. The majority of cases are considered some of the most severe forms of drug eruption. Others are associated with viral and mycoplasmal infections.
(f)	Non-ADE	ADE	Figure 10 shows a clinical course. According to the reporting system, ~25% (92/372) of all drugs causing TdP^g^ in the past five years were new quinolones (mainly levofloxacin).
(g)	Non-ADE	Non-ADE	Case: 70-year-old man with a history of hypertension. Right eye pain appeared on day X and was accompanied by blurred vision.
(h)	Non-ADE	Non-ADE	Case: 79-year-old female. The patient had been taking prednisolone 60 mg and methotrexate 6 mg for 6 months following a diagnosis of middle vasculitis.

^a^ADE: ADE suggesting.

^b^MTX: methotrexate.

^c^ABPC/SBT: ampicillin/sulbactam.

^d^CEZ: cefazolin.

^e^RA: rheumatoid arthritis.

^f^Non-ADE: Non-ADE suggesting.

^g^TdP: torsades de pointes.

Cases (c) and (d) are examples wherein the information in the previous sentence was required in order to classify the sentences. Each example would not be regarded as an ADE-suggesting sentence if only the following sentence was considered. However, when the previous sentence was also considered, the following sentence was regarded as ADE suggesting because the symptom mentioned in it may refer to an ADE caused by the drug mentioned in the previous sentence. Classification errors occurred when we added the features of the previous and following sentences.

Cases (e) and (f) are examples wherein the general statement is confused with an actual case. The corresponding sentence of each example describes the general disease caused by the drug. However, the general statement and the actual case are similar in terms of their expressions. Consequently, errors resulted.

### Limitations

Although our system detects ADE-containing articles with high precision and recall, the performance of ADE-suggesting sentence extraction is relatively poor. There are 3 possible reasons for this poor performance. The first reason is the range of context. Our system can only consider the context for 2 consecutive sentences, and this may increase false negatives. To detect ADEs within a wider context, other approaches would be required such as paragraph classification and sequential labeling, for example, CRF and a hidden Markov model.

**Figure 3:**
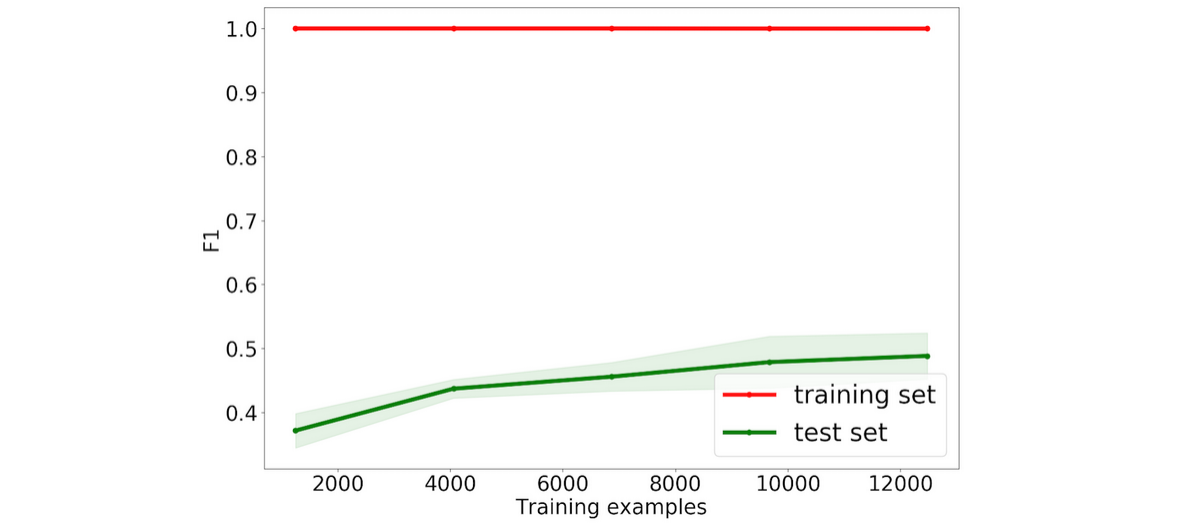
Training data size versus F1 score in ADE-suggesting sentence extraction. ADE: adverse drug event.

The second possible reason is overfitting. Figure 3 shows the F1 scores for several training data sizes. All training set F1 scores were set to 100%. By contrast, those for the validation set were low. This overfitting occurred because the training data size was small relative to the model complexity. In ADE-suggesting sentence extraction, we used 3 times as many features as the ADE-containing article extraction because of the contextual features. Although contextual features contained large amounts of information, most of them were about irrelevant words, which might lead to overfitting.

The third possible reason for the poor performance of ADE-suggesting sentence extraction is OCR error. OCR may omit and misread letters, characters, and words, thereby enlarging the vocabulary. It is expected that the improvement of OCR accuracy for Japanese scientific articles consisting of multiple columns will expedite and facilitate preprocessing.

### Comparison With Prior Studies

Numerous studies have already identified ADEs reported in medical articles via NLPs [8,9], electronic health records [5,6], and social media posts [10,11]. An annotated corpus for the automatic detection of ADEs was created for case reports in medical articles [26]. Several studies have attempted to detect ADEs by using this corpus [23,27,28]. Various approaches may be used to detect ADEs. However, relation and entity classification were mainly used for this purpose in previous studies. By contrast, our approach is based on document and sentence classification.

- Relation Classification: This approach extracts the relationship between the drug and its corresponding ADE [29,30]. Although it is the most precise way to capture these associations, it entails extensive annotations of all drugs, diseases, and their relationships, which is an expensive process. The classification of drug–disease relationships is difficult when the parameters are only remotely associated [6].
- Entity Classification: This approach focuses unilaterally on ADEs using text written about specific drugs. Diseases are classified only if they are ADEs [11,30]. This approach reduces annotation costs. By contrast, it provides no indication of ADE triggers.
- Sentence Classification: This approach detects ADE-related sentences but does not handle entities. Thus, their relationships to particular drugs are not clarified. The drug and its corresponding ADE appear mainly within a sentence [24]. Nevertheless, if the drug and its corresponding ADEs are separated by more than 1 sentence, this approach would not capture the relationship between them.
- Document Classification: This approach makes ADE-positive or ADE-negative identifications at the document level. In most cases, a document may contain multiple ADEs referred only within that document and all ADEs may be considered simultaneously. However, the output furnishes limited information and manual detection of the ADEs in all sentences is required.

Each of these approaches has both advantages and disadvantages in terms of annotation cost, coverage, and task difficulty. The relation and entity classification methods provide precise information concerning ADEs but their annotation costs are very high. This constraint severely limits their utility for minor languages such as Japanese because comparatively few medical experts are fluent in them. By contrast, document and sentence classification may be conducted at relatively low annotation costs. However, they only detect global phenomena and provide comparatively little information about ADEs. To compensate for the shortcomings of each of these approaches, our system integrated both document and sentence classification.

### Conclusions

Here, we developed a system that monitors medical articles for Japanese postmarketing surveillance. Our novel approach, which is based on both document and sentence classification, identifies articles related to ADEs and provides ADE-suggesting sentences. As our system implements a simple classification algorithm, it can be easily applied and managed in-house by pharmaceutical companies.

Our experimental results demonstrate that our system accurately extracts articles related to ADEs. It uses NLP technology which may alleviate some of the manual labor in Japanese pharmaceutical companies.

We aim to apply this system in real-world postmarketing surveillance and evaluate its efficiency and effectiveness in actual monitoring. Going forward, we will explore more complex classification algorithms that can detect a wider range of ADEs.

## Appendix

Multimedia Appendix 1 Frequency of the drugs appearing in the data.

Multimedia Appendix 2 Features and coefficients for ADE-containing article extraction.

Multimedia Appendix 3 Features and coefficients for ADE-suggesting sentence extraction.

## Abbreviations

ADE: adverse drug event
ADR: adverse drug reaction
CRF: conditional random fields
NLP: natural language processing
NN: neural network
OCR: optical character recognition

## References

[1] World Health Organization (1960). International Drug Monitoring: The Role of the Hospital (Report of a WHO Meeting).

[2] Howard RL, Avery AJ, Slavenburg S, Royal S, Pipe G, Lucassen P, Pirmohamed M (2007). Which drugs cause preventable admissions to hospital? A systematic review. Br J Clin Pharmacol.

[3] Rogers AS (1987). Adverse drug events: identification and attribution. Drug Intell Clin Pharm.

[4] Talbot JCC, Nilsson BS (1998). Pharmacovigilance in the pharmaceutical industry. Br J Clin Pharmacol.

[5] Henry S, Buchan K, Filannino M, Stubbs A, Uzuner O (2020). 2018 n2c2 shared task on adverse drug events and medication extraction in electronic health records. J Am Med Inform Assoc.

[6] Li F, Liu W, Yu H (2018). Extraction of Information Related to Adverse Drug Events from Electronic Health Record Notes: Design of an End-to-End Model Based on Deep Learning. JMIR Med Inform.

[7] Usui M, Aramaki E, Iwao T, Wakamiya S, Sakamoto T, Mochizuki M (2018). Extraction and Standardization of Patient Complaints from Electronic Medication Histories for Pharmacovigilance: Natural Language Processing Analysis in Japanese. JMIR Med Inform.

[8] Gurulingappa H, Mateen-Rajput A, Toldo L (2012). Extraction of potential adverse drug events from medical case reports. J Biomed Semantics.

[9] P Tafti A, Badger J, LaRose E, Shirzadi E, Mahnke A, Mayer J, Ye Z, Page D, Peissig P (2017). Adverse Drug Event Discovery Using Biomedical Literature: A Big Data Neural Network Adventure. JMIR Med Inform.

[10] Chen X, Faviez C, Schuck S, Lillo-Le-Louët A, Texier N, Dahamna B, Huot C, Foulquié P, Pereira S, Leroux V, Karapetiantz P, Guenegou-Arnoux A, Katsahian S, Bousquet C, Burgun A (2018). Mining Patients' Narratives in Social Media for Pharmacovigilance: Adverse Effects and Misuse of Methylphenidate. Front Pharmacol.

[11] Nikfarjam A, Sarker A, O'Connor K, Ginn R, Gonzalez G (2015). Pharmacovigilance from social media: mining adverse drug reaction mentions using sequence labeling with word embedding cluster features. J Am Med Inform Assoc.

[12] Hans M, Gupta SK (2018). Comparative evaluation of pharmacovigilance regulation of the United States, United Kingdom, Canada, India and the need for global harmonized practices. Perspect Clin Res.

[13] Food and Drug Administration (2010). Investigational New Drug Safety Reporting Requirements for Human Drug and Biological Products and Safety Reporting Requirements for Bioavailability and Bioequivalence Studies in Humans.

[14] European Commission (2011). Communication from the Commission – Detailed Guidance on the Collection, Verification and Presentation of Adverse Event/Reaction Reports Arising from Clinical Trials on Medicinal Products for Human Use (‘CT-3’).

[15] Pharmaceuticals and Medical Devices Agency Reports of Side Effects, Infectious Diseases and Defects Based on the Pharmaceutical and Medical Devices Act (for Medical Personnel) (in Japanese).

[16] NTTDATA NJK Corporation WinReader PRO v.15.0.

[17] Cohen J (1960). A Coefficient of Agreement for Nominal Scales. Educational and Psychological Measurement.

[18] Aramaki E, Yano K, Wakamiya S (2017). MedEx/J: A One-Scan Simple and Fast NLP Tool for Japanese Clinical Texts. Stud Health Technol Inform.

[19] Ito K, Nagai H, Okahisa T, Wakamiya S, Iwao T, Aramaki E (2018). J-MeDic: A Japanese disease name dictionary based on real clinical usage.

[20] Social Computing Laboratory, Nara Institute of Science and Technology HYAKUYAKU dictionary..

[21] Aramaki E, Imai T, Miyo K, Ohe K (2008). Orthographic disambiguation incorporating transliterated probability.

[22] Belinkov Y, Glass J (2019). Analysis Methods in Neural Language Processing: A Survey. Transactions of the Association for Computational Linguistics.

[23] Pedregosa F, Varoquaux G, Gramfort A (2011). Scikit-learn: Machine learning in Python. J Mach Learn Res.

[24] Negi K, Pavuri A, Patel L, Jain C (2019). A novel method for drug-adverse event extraction using machine learning. Informatics in Medicine Unlocked.

[25] Kudo T MeCab: Yet Another Part-of-Speech and Morphological Analyzer.

[26] Gurulingappa H, Rajput AM, Roberts A, Fluck J, Hofmann-Apitius M, Toldo L (2012). Development of a benchmark corpus to support the automatic extraction of drug-related adverse effects from medical case reports. J Biomed Inform.

[27] Kang N, Singh B, Bui C, Afzal Z, van MEM, Kors JA (2014). Knowledge-based extraction of adverse drug events from biomedical text. BMC Bioinformatics.

[28] Henriksson A, Kvist M, Dalianis H, Duneld M (2015). Identifying adverse drug event information in clinical notes with distributional semantic representations of context. J Biomed Inform.

[29] Zhao J, Henriksson A, Asker L, Boström H (2015). Predictive modeling of structured electronic health records for adverse drug event detection. BMC Med Inform Decis Mak.

[30] Zhao J, Henriksson A, Asker L, Bostrom H (2015). Detecting adverse drug events with multiple representations of clinical measurements.

